# VARPRISM: incorporating variant prioritization in tests of *de novo* mutation association

**DOI:** 10.1186/s13073-016-0341-9

**Published:** 2016-08-25

**Authors:** Hao Hu, Hilary Coon, Man Li, Mark Yandell, Chad D. Huff

**Affiliations:** 1 Department of Epidemiology, The University of Texas M.D. Anderson Cancer Center, Houston, TX USA; 2 Department of Psychiatry, University of Utah, Salt Lake City, UT USA; 3 Department of Human Genetics and USTAR Center for Genetic Discovery, University of Utah, Salt Lake City, UT USA

**Keywords:** *De novo* mutations, Autism spectrum disorder, Simons Simplex Collection, Likelihood ratio test, Variant prioritization

## Abstract

**Background:**

Patients with certain genetic diseases, such as autism spectrum disorder, have increased rates of *de novo* mutations within some protein-coding genes.

**Results:**

We introduce the VARiant PRIoritization SuM (VARPRISM), a software package which incorporates functional variant prioritization information to improve the power to detect *de novo *mutations influencing disease risk. VARPRISM evaluates the consequence of any given exonic mutation on the protein sequence to estimate the likelihood that the mutation is benign or damaging and conducts a likelihood ratio test on the gene level. We analyzed the Simons Simplex Collection of 2508 parent-offspring autism trios using VARPRISM, replicating 44 genes previously implicated in autism susceptibility and identifying 20 additional candidate genes, including *MYO1E, KCND3, PDCD1, DLX3,* and *TSPAN4* (false discovery rate < 0.3).

**Conclusion:**

By incorporating functional predictions, VARPRISM improved the statistical power to identify *de novo* mutations increasing disease risks. VARPRISM is available at http://www.hufflab.org/software/VARPRISM.

**Electronic supplementary material:**

The online version of this article (doi:10.1186/s13073-016-0341-9) contains supplementary material, which is available to authorized users.

## Background

*De novo* mutations contribute substantially to the risk of several genetic diseases, including Autism Spectrum Disorder (ASD) [1–3], intellectual disability, and schizophrenia [4–11]. To identify associations between *de novo* mutations and disease risk, most existing approaches separately consider different class of mutations, for example, loss-of-function mutations [12], missense mutations [12], or mutations predicted to be damaging [13]. The observed number of mutations in a gene is compared to the expected number based on the known mutation rate. A *p* value is then calculated as the tail-probability of the expected distribution under the null, derived either through Poisson approximations or simulations [12, 14]. This framework, however, suffers from a difficult optimization problem: including too many mutation classes will decrease the signal-to-noise ratio, while including too few may exclude many causal mutations. For example, in a previous report estimating the proportion of missense and likely gene disrupting (LGD) mutations contributing to ASD [12], excluding missense mutations would eliminate 57 % of causal mutations, while including missense mutations would decrease the signal-to-noise ratio from 0.75 to 0.22.

Variant prediction algorithms, such as SIFT [15], Polyphen-2 [16], and MutationTaster [17], assess the potential functional impact of genetic variants on proteins using a variety of information sources, including amino acid substitution (AAS), protein structural information, and phylogenetic conservation. These algorithms are well suited for classification of individual disease-causing variants [16]; however, because classification accuracy rarely exceeds 75 % [18], a binary inclusion-exclusion approach based on the predicted severity of each mutation will exclude many true positives.

To account for uncertainty in variant effect prediction, rare variant association tests such as SKAT [19] and VAAST [20] often use prediction scores as a continuously scaled weight in various forms, e.g. as the variance of the random effect [19] or as the likelihood in the composite likelihood ratio test [20]. A loss-of-function mutation typically receives the highest weight and therefore has the largest influence on the statistical test, while a synonymous mutation often receives a weight of 0. Previously, Jiang et al. [21] developed the fitDNM *de novo* mutation load test, which incorporates functional predictions from variant classifiers into the *de novo* mutation load test. fitDNM requires specification of probabilities for any given mutation in the protein being damaging, which can be generated, for example, using PolyPhen-2. Jiang et al. demonstrated that fitDNM has higher statistical power compared to existing methods such as Poisson tests and TADA-denovo [13].

Here we present the VARiant PRIoritization SuM (VARPRISM) software package, which predicts the functional impact of *de novo* mutations and incorporates these quantitative predictions using a likelihood ratio test to evaluate evidence of de novo mutation load. We compared the power of VARPRISM to fitDNM and Poisson tests with two simulated datasets. We then analyzed 2508 parent-offspring autism trios from the Simons Simplex Collection (SSC) to identify autism candidate genes.

## Implementation

### Statistical model of VAPRISM

VARPRISM analyzes *de novo* mutations in the genomes of affected individuals to identify genes with elevated de novo mutation rates of functional protein-coding mutations. Let *AAS*_*ij*_ be the random variable vector describing functional consequence of the *j*-th *de novo* mutation within the *i*-th affected individual in the protein of interest. *AAS*_*ij*_ includes a categorical variable (the amino acid substitution caused by the mutation, e.g. Leucine to Proline change) and a continuous variable (the PhastCons score at the mutation [22]). We use the random variable *M*_*ij*_ to denote the event that the *j*-th mutation within individual *i* occurred in the gene of interest. *M*_*ij*_ can be partitioned into two disjoint events: (1) a disease-causal mutation occurred (denoted by random variable *D*_*ij*_); and (2) a neutral mutation occurred (denoted by random variable *N*_*ij*_). For any observed *de novo* mutation, indexed by *i* and *j*, the joint probability of the *M*_*ij*_ and *AAS*_*ij*_ is:

$$ \begin{array}{l} \Pr \left({M}_{ij}, AA{S}_{ij}\left|r,m\right.\right)\\ {}= \Pr \left({N}_{ij}, AA{S}_{ij}\left|r,m\right.\right)+ \Pr \left({D}_{ij}, AA{S}_{ij}\left|r,m\right.\right)\\ {}=P\left({N}_{ij}\left|r,m\right.\right) \Pr \left( AA{S}_{ij}\left|{N}_{ij},r,m\right.\right)+P\left({D}_{ij}\left|r,m\right.\right) \Pr \left( AA{S}_{ij}\left|{D}_{ij},r,m\right.\right)\\ {}=P\left({N}_{ij}\left|r,m\right.\right)\left( AA{S}_{ij}\left|{N}_{ij}\right.\right)+P\left({D}_{ij}\left|r,m\right.\right) \Pr \left( AA{S}_{ij}\left|{D}_{ij}\right.\right)\\ {}=m \Pr \left( AA{S}_{ij}\left|{N}_{ij}\right.\right)+rm \Pr \left( AA{S}_{ij}\left|{D}_{ij}\right.\right)\end{array} $$

where *m* is the risk-neutral mutation rate (per generation per base pair) [23] at the corresponding nucleotide, which we used the average risk-neutral mutation rate in the gene of interest to approximate. *r* is the relative mutation rate of disease-causing mutations to risk-neutral mutations. The third equality holds because the distribution of *AAS*_*ij*_ is independent of the mutation rates conditional on the disease-risk.

For all *k*_*i*_ observed mutations in affected individual *i*, the joint probability is:

$$ \begin{array}{l} \Pr \left({M}_{i\kern.1em \cdotp }, AA{S}_{i\kern.1em \cdotp}\left|r,m\right.\right)\\ {}={\displaystyle \prod_{j=1}^{k_i} \Pr \left({M}_{ij}, AA{S}_{ij}\left|r,m\right.\right)}\\ {}={\displaystyle \prod_{j=1}^{k_i}\left[m \Pr \left( AA{S}_{ij}\left|{N}_{ij}\right.\right)+rm \Pr \left( AA{S}_{ij}\left|{D}_{ij}\right.\right)\right]}.\end{array} $$

The first equality holds under the assumption that *de novo* mutations occur independently, conditional on the neutral and disease-causal de novo mutation rates. Under the null (*r* = 0), this assumption is strictly satisfied; under the alternative, this is justified because the mutation rate is sufficiently low to be very unlikely to observe two *de novo* mutations in the same gene of the same individual.

To account for genomic sites where no *de novo* mutations occurred in our model, we use *G*_*i*•_ to denote the genotype of individual *i*, with the following density function:

$$ \begin{array}{l} \Pr \left({G}_{i\kern.1em \cdotp }, AA{S}_{i\kern.1em \cdotp}\left|r,m\right.\right)\\ {}={\left(1-m-rm\right)}^{n-{k}_i}{\displaystyle \prod_{j=1}^{k_i}\left[m \Pr \left( AA{S}_{ij}\left|{N}_{ij}\right.\right)+rm \Pr \left( AA{S}_{ij}\left|{D}_{ij}\right.\right)\right]}\end{array} $$

where *n* is 2 times the number of base pairs in the coding sequence of the gene of interest. If we use ***G*** and ***AAS*** to denote the genotypes and functional impact of amino acid substitutions in all affected individuals and assume independence across individuals, then the following holds:

$$ \begin{array}{l} \Pr \left(\boldsymbol{G},\boldsymbol{A}\boldsymbol{A}\boldsymbol{S}\left|r,m\right.\right)\\ {}={\displaystyle \prod_{i=1}^t\left\{{\left(\mathrm{l}-m-rm\right)}^{n-{k}_i}{\displaystyle \prod_{j=1}^{k_i}\left[m \Pr \left( AA{S}_{ij}\left|{N}_{ij}\right.\right)+rm \Pr \left( AA{S}_{ij}\left|{D}_{ij}\right.\right)\right]}\right\}}\end{array} $$

where *t* is the number of affected individuals. The parameter *m* can be estimated either from literature or from the data. The quantity above is equivalently the likelihood of the parameter *r* (that is, L(*r*)).

Under the null hypothesis (H_0_), no *de novo* mutations within the gene of interest contribute to disease risk. This implies that the probability of having a disease-causal *de novo* mutation is 0, or formally, Pr(*D*_*ij*_|*r*,*m*) = 0 for all values of *i* and *j*. Given Pr(*D*_*ij*_|*r,m*) = *rm* and *m* ≠ 0, we have *r* = 0. Conversely, *r* = 0 suggests that the relative mutation rate of disease-causing mutations to risk-neutral mutations is 0 (by definition), and therefore no disease-causing *de novo* mutation can occur. We can apply a likelihood ratio test to calculate the following test statistic:

$$ D=-2\left[ \ln \left( \Pr \left(\boldsymbol{G},\boldsymbol{A}\boldsymbol{A}\boldsymbol{S}\Big|r=0,m\right)\right)-{ \sup}_r\left\{ \ln \Pr \left(\boldsymbol{G},\boldsymbol{A}\boldsymbol{A}\boldsymbol{S}\Big|r,m\right)\right\}\right] $$

To obtain the MLE of *r* under the alternative model, VARPRISM uses the Newton–Raphson method. Specifically, we first performed the following transformation on the variable *r*: *r* = *e*^*t*^. We used the following initial values of *t*: log(0.01), log(0.1), log(1), log(5), log(10), log(20), and log(100), and performed the maximization procedures on each initial value. Each time, *t* is iteratively updated with Newton step $\left({t}^{+}=t-{\left(\frac{d^2 \log \Pr \left(\boldsymbol{G},\boldsymbol{A}\boldsymbol{A}\boldsymbol{S}\Big|t,m\right)}{d{t}^2}\right)}^{-1}\frac{d \log \Pr \left(\boldsymbol{G},\boldsymbol{A}\boldsymbol{A}\boldsymbol{S}\Big|t,m\right)}{dt}\right)$ until the maximal number of iterations (by default 20) were performed. At the end, the *t* value that generated the maximum log-likelihood was selected to calculate *r* using *r* = *e*^*t*^. To calculate the statistical significance of the observed *D*, VARPRISM uses Monte-Carlo methods to simulate mutations in the gene of interest conditioned on the local mutation rate of the gene. Assuming *n*_*1*_ out of *n*_*2*_ total simulations have a value of *D* no less than the observed *D*, then the *p* value is calculated as (*n*_*1*_ + 1)/(*n*_*2*_ + 1).

### Mutational model

A correct estimation of local mutation rate is essential for robust statistical characterization of genes with disease-causing *de novo* mutations. To control for heterogeneity in local mutation rates, VARPRISM incorporates the mutation rate estimate reported by Francioli et al. [23]. Specifically, Francioli et al. estimated the empirical distribution of genome-wide mutation rates from 250 parent-offspring families, accounting for flanking sequence context, local mutation rates, mutation type, and the transcribed strand [23]. Based on this estimate and the actual nucleotide sequence, we summed the mutation rates at every base pair of the gene to derive the expected *de novo* mutation rate (*m*) for each protein-coding gene. Similarly, we compute the distribution of amino acid substitutions caused by mutations under the null model (Pr(AAS|N)), by considering all possible mutations at each base pair within the exons of the gene. Pr(AAS|N) and *m* are used in formula (1) and in Monte-Carlo simulations. Under the alternative model, the distribution of amino acid substitutions (Pr(AAS|D)) is estimated by the AAS frequency spectrum observed in the Human Gene Mutation Database (HGMD) [24]. This approach (CASM) was previously used to calculate functional weights of rare variants in gene-based association tests; the details on the training process of CASM were described in the Additional file 1: Supplementary Methods and in our previous publication [18].

Note that the calculation of Pr(AAS|N) is exact assuming the mutation rate reported in [23] is accurate. However, Pr(AAS|D) is often only an approximation, given that the AAS distribution for all possible mutations that could influence the risk of a specific disease within a given gene cannot be known for *ab initio* risk gene discovery. When Pr(AAS|D) is correctly specified, then the likelihood ratio test statistic is approximately distributed as a 50:50 mixture of two chi-square variables with 0 and 1 degree(s) of freedom, respectively [25]. Otherwise, under the null, the likelihood becomes:

$$ \ln L\left(r=0\right)= \ln \left(\mathrm{l}-m\right){\displaystyle \sum_{i=1}^t\left(n-{k}_i\right)+}{\displaystyle \sum_{i=1}^t{\displaystyle \sum_{j=1}^{k_i} \ln \left[m \Pr \left( AA{S}_{ij}\left|{N}_{ij}\right.\right)\right]}} $$

which does not depend on Pr(AAS|D). Under the alternative, the maximal likelihood is:

$$ \begin{array}{l} \sup \left\{ \ln L(r):r\ge 0\right\}\\ {}= \ln \left(\mathrm{l}-m-\widehat{r}m\right){\displaystyle \sum_{i=1}^t\left(n-{k}_i\right)+{\displaystyle \sum_{i=1}^t{\displaystyle \sum_{j=1}^{k_i} \ln \left[m \Pr \left( AA{S}_{ij}\left|{N}_{ij}\right.\right)+\widehat{r}m \Pr \left( AA{S}_{ij}\left|{D}_{ij}\right.\right)\right]}}}\end{array} $$

which is a function of Pr(AAS|D). In other words, although the null model likelihood is correctly specified, the alternative model likelihood is often not. In this scenario, as Vuong has demonstrated, the likelihood ratio test still applies (theorem 7.2. in [26]), but the distribution of the test statistic becomes a linear combination of χ^2^ variables whose weights depend on the likelihood in the alternative model. Since Pr(AAS|D) is unknown, by default VARPRISM avoids the analytical approximation for the distribution of the test statistic, instead relying on Monte-Carlo simulations to sample from its null distribution. Because the Monte-Carlo simulations do not use Pr(AAS|D) to generate mutations, a Type I error rate is correctly specified (Table 1).

**Table 1:** Type I error of VARPRISM in the null simulations (showing 95 % CI in the bracket)

	Autism		HGMD	
	alpha = 0.05	alpha = 0.01	alpha = 0.05	alpha = 0.01
VARPRISM	0.0499 (0.0456–0.0545)	0.0110 (0.0090–0.0133)	0.0482 (0.0440–0.0527)	0.0104 (0.0085–0.0126)
fitDNM	0.0510 (0.0468–0.0555)	0.0088 (0.0070–0.0108)	0.0470 (0.0429–0.0514)	0.0096 (0.0078–0.0117)
Poisson-all	0.0219 (0.0191–0.0250)	0.0077 (0.0061–0.0096)	0.0213 (0.0185–0.0244)	0.0075 (0.0059–0.0094)
Poisson-LGD	0.0265 (0.0234–0.0299)	0.0018 (0.0011–0.0028)	0.0240 (0.0211–0.0272)	0.0025 (0.0016–0.0037)

Since the observed *de novo* mutation rate may depend on factors that differ across studies (for example, paternal age at conception and variant calling procedures), we provided a script to calibrate the genome-wide average *de novo* mutation rate based on the frequency of *de novo* mutations in the current dataset. The *de novo* mutation rate of small insertions and deletions (indels) are estimated separately from SNVs, because the ratio of indel and SNV mutations will likely vary by the sequencing platform and variant calling pipelines [27]. The estimated SNV and indel mutation rates are required parameters in VARPRISM. When indels are not of interest, the indel mutation rate can be set to 0.

The mutation simulation framework in VARPRISM is also implemented into the pVAAST software package v2.2 [28], which can jointly analyze pedigrees with *de novo* mutations, inherited variants, and variants in sporadic cases. pVAAST incorporates gene-based linkage analysis, case-control association, and variant prediction information to identify genes contributing to disease risk in pedigrees.

### Power analyses

**Fig. 1:**
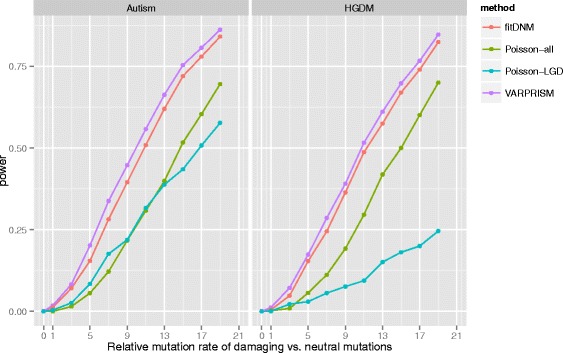
Power comparison between fitDNM, Poisson-all, Poisson-LGD, and VARPRISM. *Left*: Power benchmark using *de novo* mutations in ASD risk genes as damaging mutations. *Right*: Power benchmark using Human Gene Mutation Database (HGMD) variants as damaging mutations. The sample size is 5000 genomes and the number of trials is 1000. We set the statistical significance threshold at 5 × 10^–4^

Power analyses in Fig. 1 were generated from simulated *de novo* mutation data. The simulated gene length was 2000 bp with a baseline mutation rate of 1.2 × 10^–8^ per generation per haploid base pair. The relative causal mutation rates (*r* in the formula 1) vary from 0 to 19. We generated the number of mutations in each simulation from a Poisson distribution, with the expected mutation count equal to the sum of baseline mutation rate multiplied by (1 + *r*). To simulate causal mutations, we sampled mutations from either HGMD mutations (excluding mutations used for training VARPRISM) or *de novo* protein-altering mutations found in ASD cases within genes with false discovery rate (FDR) < 0.01 in [29]. We note that these two sets of mutations (HGMD mutations and ASD *de novo* mutations from [29]) do not have any overlap. To simulate risk-neutral protein-coding mutations, we sampled mutations from possible protein-coding mutations in the human exome. The probability of sampling was proportional to corresponding mutation rates. For each simulated mutation, the type of nucleotide and amino acid substitution, local mutation rate, Phastcons score [22], and Polyphen-2 score [16] were the same as the source mutation in the exome. All simulations used a sample size of 5000 cases with the exception of the case of *r* = 0 (null simulation), which used a sample size of 100,000 cases to detect any potential inflation or deflation of type I error. For each *r* > 0, 1000 repetitions were generated. At *r* = 0, 10,000 repetitions were generated.

To accommodate fitDNM, we created customized scripts to generate necessary input files of fitDNM from simulated data. Since fitDNM currently does not currently support indels, we did not include indels in our simulations.

## Results

### Overview of VARPRISM

VARPRISM tests the null hypothesis that *de novo* mutations in a gene are not associated with disease. We derived the log-likelihood of the observed *de novo* mutations and their corresponding amino acid substitutions (AAS). VARPRISM calculates the AAS likelihoods using a conservation-controlled amino acid substitution matrix (CASM), which was introduced in VAAST 2.0 [18]. CASM is a variant prediction algorithm that incorporates AAS and phylogenetic conservation information and is calibrated using disease mutation databases (Additional file 1: Supplementary Methods). To illustrate the ability of the CASM score to differentiate between benign and disease-causing mutations, we calculated CASM scores for each *de novo* mutation in 15 previously reported ASD risk genes [30] in an ASD whole-exome sequencing study [29]. For comparison, we randomly generated 1000 single nucleotide *de novo* mutations in these genes according to the actual genomic sequences and performed the same calculation. The mean CASM score (likelihood of AAS under alternative divided by null) of ASD mutations was 1.83, compared to 1.13 for randomly generated mutations (*p* = 6.4 × 10^–4^, Wilcoxon rank sum test).

To conduct a gene-based test of *de novo* mutation load, VARPRISM estimates the relative mutation rate of disease-causing mutations (*r*) among individuals with the disease compared to a baseline mutation rate in each gene. Under the null hypothesis that *de novo* mutations in a gene are not associated with the disease phenotype, *r* is equal to 0, indicating that the mutation rate among affected individuals is the same as the baseline rate. VARPRISM then calculates the likelihood ratio statistics using the maximum likelihood estimate (MLE) of *r* and evaluates statistical significance via Monte-Carlo simulations. The details of the statistical model are elaborated in the “Implementation” section.

To control for mutation rate heterogeneity resulting from flanking sequence context, DNA replication start sites, nucleotide composition, etc., VARPRISM incorporates site-specific mutation rate estimates throughout the genome. By default, VARPRISM uses the mutation rate estimates from Francioli et al. [23], although alternative estimates can be provided. The mutation simulation pipeline used by VARPRISM is also implemented in pVAAST v2.2 [28], which can jointly analyze *de novo* and inherited mutations in pedigrees.

### Power in simulations

To evaluate the statistical power of VARPRISM, we simulated mutation data for 5000 parent-offspring trios using mutations from the HGMD [24] or the 13 genes with FDR < 0.01 in [29] based on an excess of *de novo* mutations identified in ASD cases, varying the relative mutation rate of damaging mutations in the simulated causal gene (see “Implementation”). We calculated power as the proportion of simulations in which we found a significant association (*α* = 5 × 10^–4^). For each simulation, we evaluated four tests: VARPRISM, fitDNM, a Poisson test using all non-synonymous mutations (Poisson-all), and a Poisson test using only likely gene-disrupting (nonsense, frameshift, and splice sites) mutations (Poisson-LGD). For fitDNM, we used Polyphen-2 to predict mutation impact as in the original fitDNM article [21] and suggested by the User Manual; however, we also explored using transformed CASM scores (Additional file 1: Supplementary Methods; Figure S1). In both simulations, the statistical power of VARPRISM was consistently higher than the other three tests (Fig. 1). In the HGMD simulation, when VARPRISM achieved 85 % power, the power of the other three methods was 82 %, 70 %, and 25 % for fitDNM, Poisson-all, and Poisson-LGD, respectively. In the ASD simulation, when VARPRISM achieved 86 % power, the power of the other three methods was 84 %, 70 %, and 58 %, for fitDNM, Poisson-all, and Poisson-LGD, respectively. The average damaging mutation rates estimated by VARPRISM closely approximated their simulated values (Additional file 1: Figure S2). The type I error rates of each method are shown in Table 1. For VARPRISM, the observed type I error rate was consistent with the nominal rate at all levels (alpha = 0.05 and 0.01).

### VARPRISM analysis of *de novo* mutations in congenital heart disease

Previously, Zaidi et al. [31] investigated the role of *de novo* mutations in the etiology of congenital heart disease (CHD) from whole-exome sequencing data on 362 CHD parent-offspring trios with affected probands and unaffected parents. Within probands, they identified eight genes with *de novo* mutations that are involved in the production, removal, or reading of H3K4 methylation (H3K4me pathway) [31]. The authors also found that the gene *SMAD2*, a regulator of H3K27 methylation, was mutated twice. VARPRISM identified *SMAD2* and all eight genes with nominal significance (*p* < 0.05). In comparison, Poisson-all, Poisson-LGD, and fitDNM identified six, four, and seven genes, respectively, with nominal significance (Additional file 1: Figure S3). *SMAD2* was genome-wide significant (*p* = 2.1 × 10^–6^) with VARPRISM but not with Poisson-all (*p* = 2.3 × 10^–5^), Poisson-LGD (*p* = 9.6 × 10^–4^), or fitDNM (*p* = 4.6 × 10^–6^). We also jointly evaluated the association of genes in the H3K4me pathway with CHD by combining *p* values for each of the four tests from all 30 genes in the H3K4me pathway using Fisher’s method [32]. VARPRISM was the only test that identified a statistically significant enrichment of genes in the H3K4me pathway, with *p* equal to 0.017 compared to 0.36, 0.95, and 0.18 by Poisson-all, Poisson-LGD and fitDNM, respectively.

### VARPRISM analysis of *de novo* mutations in ASD

The SSC, established by the Simons Foundation Autism Research Initiative (SFARI), is a genetic resource for autism studies that includes samples from thousands of simplex pedigrees with one affected child and unaffected parents and siblings [12, 33, 34]. Previously, Iossifov et al. [12] described results from whole-exome sequencing of 2,508 affected children from the SSC dataset. In this study, they identified 391 *de novo* LGD mutations in 353 genes, among which 27 target genes contain recurrent LGD mutations. They also identified 2801 missense mutations within 1500 genes, although the original study focused on analyzing LGD mutations [12]. We perform a joint analysis of LGD and missense mutations using VARPRISM, fitDNM, Poisson-all, and Poisson-LGD to identify additional candidate ASD risk genes [12, 33, 34]. Note that the current implementation of fitDNM does not support indels, and thus only single nucleotide variants were included in the fitDNM analysis.

With an FDR of 0.1, VARPRISM identified 19 genes, compared to four by fitDNM, four by Poisson-all, and three by Possion-LGD (Table 2). Of the 19 candidate genes identified by VARPRISM, 17 were in genes previously reported to harbor *de novo* mutations in ASD cases (Additional file 1: Table S1). With one exception (*PTEN*), all the candidate genes found by fitDNM, Poisson-all, and Poisson-LGD were identified by VARPRISM. We then tested enrichment among genes in AutDB, which is a large collection of ASD candidate risk genes created by first performing data-mining on published scientific articles and then manually annotating each gene entry by expert biologists [35]. Four types of genes were collected in AutDB: genes implicated in rare monogenic forms of ASD; genes implicated in syndromic forms on autism; genes that carry a relatively small risk for ASD identified in genetic association studies; and genes previously reported to be functionally relevant to ASD biology. Fifteen out of the 19 genes identified by VARPRISM at an FDR of 0.1 were present in AutDB (0.7 expected by chance; *p* < 2.2 × 10^–16^), including: *DYRK1A*, *WAC*, *TBL1XR1*, *KDM6B*, *GRIN2B*, *DSCAM*, *POGZ*, *SCN2A*, *SUV420H1*, *CHD8*, *TBR1*, *KDM5B*, *KATNAL2*, *TCF7L2*, and *CHD2*.

**Table 2:** List of genes identified by VARPRISM, fitDNM, Poisson-all, and Poisson-LGD in the SSC dataset

	FDR 0.1	FDR 0.3
VARPRISM	*CHD8*, *DYRK1A*, *SCN2A*, *GRIN2B*, *POGZ*, *SUV420H1*, *KDM5B*, *TBR1*, *KATNAL2*, *MYH10*, *TCF7L2*, *TBL1XR1*, *DSCAM*, *KDM6B*, *OR10Z1*, *CHD2*, *WAC*, *PDCD1*, *MFRP*	*CHD8*, *DYRK1A*, *SCN2A*, *GRIN2B*, *POGZ*, *SUV420H1*, *KDM5B*, *TBR1*, *KATNAL2*, *MYH10*, *TCF7L2*, *TBL1XR1*, *DSCAM*, *KDM6B*, *OR10Z1*, *CHD2*, *WAC*, *PDCD1*, *MFRP*, *SLC6A8*, *FOXP1*, *ANK2*, *PPP2R5D*, *ZC3H4*, *ARID1B*, *KCND3*, *ADNP*, *KRTAP4-4*, *ZNF555*, *PTEN*, *CTCF*, *USP45*, *MYO1E*, *DNMT3A*, *DIP2A*, *GLRA2*, *SYNGAP1*, *NCKAP1*, *MPP6*, *NR3C2*, *ELAVL3*, *PLEKHA8*, *PTK7*, *TSPAN4*, *TERF2*, *GIGYF1*, *PHF2*, *MLL5*, *TSR2*, *S100G*, *AKR1C2*, *SLC6A1*, *MED13L*, *BST2*, *C2orf42*, *GLI4*, *DLX3*, *NUDT4*, *CTNNB1*, *RIPPLY1*, *NTNG1*, *DCAF4L2*, *PAFAH1B2*, *TUBGCP4*
fitDNM	*SCN2A*, *PTEN*, *SUV420H1*, *KDM5B*	*SCN2A*, *PTEN*, *SUV420H1*, *KDM5B*, *SLC6A1*, *PTK7*, *PRB4*
Poisson-all	*CHD8*, *SCN2A*, *DYRK1A*, *PTEN*	*CHD8*, *SCN2A*, *DYRK1A*, *PTEN*, *POGZ*
Poisson-LGD	*CHD8*, *DYRK1A*, *GRIN2B*	*CHD8*, *DYRK1A*, *GRIN2B*, *CHD2*, *DSCAM*, *KATNAL2*, *TCF7L2*, *WAC*, *ANK2*, *FOXP1*

**Fig. 2:**
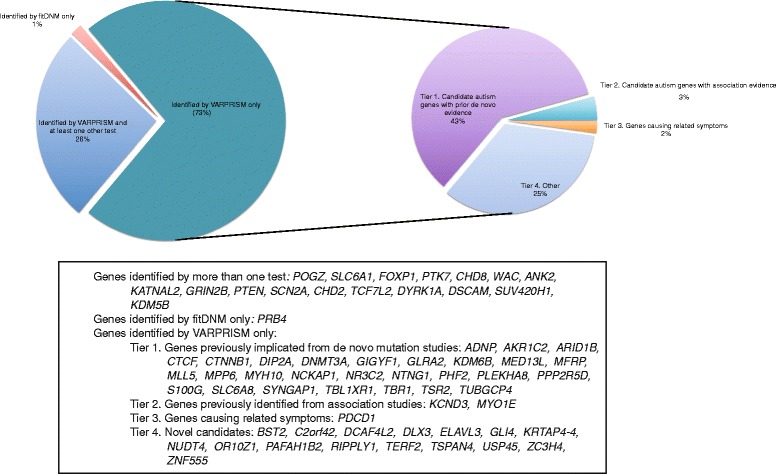
Candidate *de novo* mutated genes identified by VARPRISM, Poisson tests, and fitDNM. *Left*, the *pie chart* shows the relative proportion of genes found by VARPRISM and any other tests (Poisson-all, Poisson-LGD, or fitDNM). *Right*, the *pie chart* further illustrates the level of evidence for genes found by VARPRISM only. Gene names in each category were listed below

Due to the sparsity of *de novo* mutations and the limited statistical power in our dataset, we also explored a more relaxed FDR threshold of 0.3, as applied previously in a recent study of *de novo* mutations in ASD [29]. VARPRISM identified 64 candidate genes, compared to seven by fitDNM, 10 by Poisson-LGD, and five by Poisson-all (Table 2 and Fig. 2). With an FDR of 0.3, the expected number of true positive ASD risk gene found by VARPRISM was 45, which was 2.6 times higher than the expected number under FDR of 0.1(17). Of the 64 genes identified by VARPRISM, 33 were present in AutDB (2.3 expected by chance; *p* < 2.2 × 10^–16^). Thus, the relaxed cutoff increased the proportion of false positive discoveries in our candidate genes, but also substantially improved the power to identify novel ASD risk genes. Excluding genes that were also identified by Poisson tests or fitDNM, 28 of the 47 remaining genes have been previously reported to contain de novo mutations among ASD cases in other datasets. Many of these were repeatedly reported to be associated with ASD, including *CTCF*, *SYNGAP1*, *SLC6A8*, *NTNG1*, and *GLRA2* (Additional file 1: Table S1). Three of the 19 remaining VARPRISM candidate genes without *de novo* association evidence, *MYO1E*, *KCND3*, and *PDCD1* are potentially promising candidates, due to their associations with social communication problem (*MYO1E*) [36], deficits in non-verbal communication (*KCND3*) [37] and 2q37-deletion syndrome (*PDCD1*) [38]. Two additional genes with FDR < 0.3, *DLX3* and *TSPAN4*, are involved in mechanisms related to ASD. We described the potential implications of these genes in ASD in more details in the “Discussion” section.

### Over-representation of candidate autism genes in functional gene classes

**Fig. 3:**
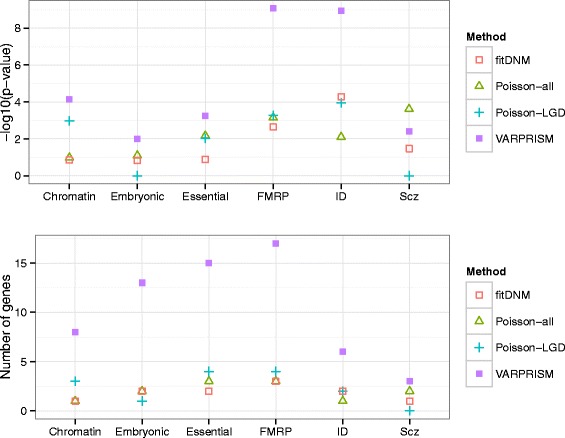
Overlap between candidate ASD genes and six functional gene classes. Four methods for identifying candidate genes were compared. The upper plot shows the p-values under the null hypothesis that the candidate ASD gene list is independent from the functional gene classes, calculated using a binomial test; the lower plot shows the actual number of overlapping genes between candidate genes and the functional gene classes

Iossifov et al. identified six functional gene classes that were significantly overrepresented among their candidate autism genes: fragile X mental retardation protein, chromatin modification, embryonic development, essential genes, schizophrenia *de novo* mutation, and intellectual disability *de novo* mutations gene classes [12]. Genes identified by VARPRISM with FDR < 0.3 from SSC were significantly over-represented in each of these six functional gene classes (Fig. 3 and Additional file 1: Table S2). In particular, VARPRISM identified six genes in the intellectual disability gene class, which was a 59.0-fold enrichment relative to expectation (*p* = 1.1 × 10^–9^). In comparison, fitDNM, Poisson-all, and Poisson-LGD identified two (*p* = 5.3 × 10^–5^), one (*p* = 7.9 × 10^–3^), and two (*p* = 1.1 × 10^–4^) genes in the intellectual disability gene class, respectively. Similarly, in the fragile X mental retardation protein gene class, VARPRISM identified 17 genes (6.3-fold enrichment; *p* = 8.5 × 10^–10^), while fitDNM identified three (*p* = 2.30 × 10^–3^), Poisson-all identified three (*p* = 7.0 × 10^–4^), and Poisson-LGD identified four (*p* = 5.4 × 10^–4^).

To search for additional functional gene classes involved in ASD, we evaluated the VARPRISM candidate gene list in the Database for Annotation, Visualization and Integrated Discovery (DAVID) (Additional file 1: Table S3). After removing closely related GO terms, the top 3 terms were GO:0016568 (chromatin modification, Benjamini FDR = 9.3 × 10^-4^), GO:0006325 (chromatin organization, Benjamini FDR = 1.5 × 10^-3^) and GO:0060070 (Wnt receptor signaling pathway through beta-catenin, Benjamini FDR = 0.047). These findings were in agreement with the autism-related functional pathways identified by Iossifov et al [12] and De Rubeis et al [29].

## Discussion

Most existing *de novo* mutation load tests classify *de novo* mutation as either damaging or neutral in a dichotomous fashion and include only the putatively damaging mutations in the test [12, 14]. However, many different criteria of choosing damaging mutations exist, for example: (1) protein-coding mutations; (2) mutations predicted as damaging by variant classification software; or (3) loss-of-function mutations. Choosing a more stringent criterion can increase the signal-to-noise ratio of the analyses but carries the risk of missing a true risk gene. Some researchers opt to perform multiple *de novo* mutation load tests with different classification criteria; doing so, however, increases the burden of multiple-testing correction and can also result in loss-of-power power for causal genes with mutations from multiple risk tiers. For most disease-gene associations, no existing variant classification scheme can generate sufficiently accurate classifications to produce an optimal dichotomous *de novo* mutation test. For example, we previously reported that SIFT, PolyPhen-2, and MutationTaster correctly predicted disease causing and benign variants only 57 %, 62 %, and 74 % of the time, respectively, on the HGMD and 1000 genomes testing sets [18], which is consistent with findings reported elsewhere [39–41]. As a result, dichotomous classification schemes will typically exclude many true disease-causing mutations. An alternative approach, adopted by TADA-denovo [13], is to analyze multiple categories of mutations (e.g. LGD and missense) separately, and then combine the Bayes factor of individual analyses. This method, however, requires classifying mutations into broad categories and cannot readily incorporate functional prediction scores generated by variant classification tools. Indeed, Jiang et al. have previously shown that a *de novo* mutation test (fitDNM) using continuously scaled functional weight outperforms TADA-denovo in simulations and in neurological-disorder datasets [21].

We have developed an alternative approach, implemented in the software package VARPRISM, which jointly considers the full likelihood of the observed mutations and the predicted impacts on protein function. By default, VARPRISM calculates the CASM score of each mutation to predict potential pathogenicity, although external variant prediction scores from other tools can alternatively be provided on the command line. VARPRISM exhibited increased statistical power for detecting *de novo* mutation disease risk genes compared to alternative approaches (fitDNM and Poisson tests) in both simulated and real datasets. VARPRISM derives baseline mutation rates under the null model from site-specific mutation rate estimates across the genome. The software defaults to the mutation rate estimates from Francioli et al. [23], but also supports user provided site-specific mutation rate estimates. Thus, VARPRISM is compatible with alternate null models that modify mutation rates under the null model to account for the estimated strength of purifying selection (i.e. level of selective constraint) [14].

VARPRISM shares a few similarities with the fitDNM test. Both tests derived the full likelihood of observed *de novo* mutations among affected individuals; both also incorporated a functional prediction term for each mutation. However, a few differences exist. First, fitDNM is based on a score test while VARPRISM is based on a likelihood ratio test. Although asymptotically equivalent [42], in practice the performances of these two tests frequently vary [43–45]. Second, fitDNM considers the posterior probability of a mutation being damaging as the functional weight (e.g. PolyPhen-2 score), while VARPRISM employs the likelihood ratio of a mutation being damaging versus risk-neutral (e.g. CASM score). Finally, although the fitDNM model can in theory analyze small indels, this functionality is not available in the current implementation, which likely accounts for the differences in performance between fitDNM and VARPRISM in the SSC dataset. For example, among the 64 genes with FDR < 0.3 in VARPRISM, 34 contained at least one *de novo* indel.

In VARPRISM, the likelihood distribution of AAS caused by damaging mutations was trained using the HGMD. While this distribution may not accurately represent the true AAS distribution for *de novo* mutations, only the alternative model is potentially affected. Thus, mis-specification of the AAS distribution for damaging mutations will not inflate Type-I error (Table 1; also see “Implementation”), but may compromise power. We explored this possibility by comparing VARPRISM’s power between our HGMD and ASD simulations. In the former dataset, the training and testing mutations were both random disjoint subsets from HGMD, and therefore is the scenario where the AAS distribution used by VARPRISM reflects the true distribution under the alternative model. In the latter dataset, the training set remained the same, but mutations in the testing set were sampled from *de novo* mutations in known ASD risk genes in cases. Interestingly, despite the mis-specified AAS distribution, the power from the HGMD simulations was consistently 3–5 % lower than in ASD simulations. This unexpected difference in power was the result of underlying differences in the proportion of LGD mutations in each dataset, which was 37 % in ASD and 19 % in HGMD. Because LGD mutations are rare under the null model, a large proportion of LGD mutations under the alternative model increased the signal-to-noise ratio in VARPRISM, resulting in a corresponding increase in statistical power. Given that purifying selection has less of an effect on the distribution of *de novo* mutations compared to inherited variation, in most disease datasets analyzed by VARPRISM, the proportion of LGD mutations will probably be higher than that of HGMD. Therefore, we expect VARPRISM to exhibit robust performance in the context of mis-specified AAS distributions in most situations.

Our VAPRISM analysis of the SSC dataset identified 64 candidate ASD risk genes (FDR < 0.3) compared to 18 from the union of the other three tests (fitDNM, Poisson-all, and Poisson-LGD). The majority (44 out of 64) of these genes were either annotated by AutDB as candidate risk genes or previously found to contain *de novo* mutations in ASD cases. Three novel VARPRISM candidate genes, *MYO1E* (q = 0.21), *KCND3* (q = 0.19), and *PDCD1* (q = 0.10), were especially promising due to their implications in autistic symptoms in previous association studies. *MYO1E* encodes a member of the myosin protein family and is involved in intracellular movement and membrane trafficking [46]. In a previous cohort study on UK population-based birth cohort [36], a common variant, rs4218 in *MYO1E*, was the top genome-wide signal associated with Short Pragmatic Composite Score (SPC), which measures social communication abilities (*p* = 2.6 × 10^–8^). Another sequencing study found mutations within the putatively regulatory regions of the *MYO1E* gene in autism-affected probands [47]. *KCND3* encodes potassium channels and functions in creating action potentials [46] and has been significantly associated with Non-Verbal Communication (NVC) score in haplotype-block based association tests of two independent samples [37]. In that study, two haplotype blocks in the introns of *KCND3* were significantly associated with NVC with the Family-Based Association Test (*p* = 0.02 and 0.0006). The third gene, *PDCD1*, is one of the genes disrupted in 2q37-deletion syndrome. The 2q37 locus is one of the most frequently deleted subtelomric region; the symptoms of its deletion include autistic phenotypes, intellectual disability, and seizures [38].

Two other VARPRISM candidate genes are potentially interesting due to the similarity of their molecular functions to known ASD risk genes. *DLX3* (q = 0.29) belongs to the distal-less homeobox gene family, which is essential in regulating forebrain and basal ganglia development [48]. Variants in both *DLX1* and *DLX2*, which are genes homologous to *DLX3*, increase the risk of ASD in a previous association study. [49] *TSPAN4* (q = 0.24) is a member of the tetraspanin family. A missense variant in *TSPAN7*, a homolog of *TSPAN4*, is associated with X-linked mental retardation [50]; the same variant was also present in an autistic child in an independent study [51]. These potential associations should be interpreted with caution given the relaxed FDR cutoff.

## Conclusion

We presented a new statistical framework and software package, VARPRISM, which incorporates variant prioritization information to identify genes with a statistically significant excess of de novo mutations contributing to genetic diseases. We applied our method to the ASD dataset in Iossifov et al. [12] and identified 64 ASD candidates risk genes with FDR < 0.3, of which 44 have previously been implicated in ASD. Our results demonstrate that incorporating AAS and phylogenetic conservation information into the statistical analyses of de novo mutations can substantially improve the power of disease gene discovery.

## Availability and requirements

VARPRISM runs under Linux or OS X environment and requires Perl and CPAN installed. The main input file of VARPRISM is a list of mutations with annotated impacts on the protein function, which can be generated with either VAAST [20] or ANNOVAR [52]. The running time for a genome-wide VARPRISM analysis on a 2500-sample dataset on an Intel Xeon 2.00 GHz CPU was 14.6 h. VARPRISM is available for download at http://www.hufflab.org/software/VARPRISM/.

## Additional file

Additional file 1: Contains: Supplementary Methods, Tables S1–S3, and Figures S1–S3. (DOCX 389 kb)
