# Supplementary material for: VARPRISM: incorporating variant prioritization in tests of de novo mutation association
Source: Genome Med. 2016 Aug 25;8(1):91. doi: 10.1186/s13073-016-0341-9 (PMC4997702; doi:10.1186/s13073-016-0341-9)
Supplement: Additional file 1: — Contains: Supplementary Methods, Tables S1–S3, and Figures S1–S3. (DOCX 389 kb) [file 13073_2016_341_MOESM1_ESM.docx]

**Supplementary Methods**

**Conservation-Controlled Amino Substitution Matrix (CASM)**

CASM calculates two probabilities: 1) the probability that we observe a given type of amino acid substitution (AAS_i_) when the corresponding genetic variant is damaging (D_i_) and 2) the probability that we observe the same AAS when the variant is neutral (N_i_). Then the CASM score is calculated as the ratio of these two probabilities. The details of CASM calculation was described previously[1]. For a simple illustration, suppose we want to predict the effect of a Leucine to Serine (L->S) mutation at a genomic position with PhastCons score [2] of 1 on the disease risk. We denote the likelihood that this mutation is neutral by *h_L->S,1_* (the subscripts indicates the type of amino acid change and the PhastCons score). We use the following heuristic formula:

$$h_{L->S,1}=\left(\sum_{j=1}^{m_{L->S}} f_{j}P_{L->S}^{j}\right)/\left(\sum_{i=j}^{m} f_{j}\right)$$

where *m_L->S_* is the total number of possible L->S mutation caused by nucleotide changes in the current gene; m is the total number of possible protein-coding mutations in the current gene; *f_j_* is the mutation rate at the j-th possible mutation; $P_{L->S}^{j}$ is the PhastCons score of the j-th possible L->S mutation. The numerator estimates the total mutation rate of L->S mutation in the current gene weighted by their corresponding PhastCons score. The denominator calculated the summed protein-coding mutation rate in the current gene.

To estimate the likelihood that an L->S mutation with PhastCons score of 1 is damaging (*a_L->S,1_*), we use the following calculation:

$$a_{L->S,1}=\left(\sum_{j=1}^{n_{L->S}} P_{L->S}^{j}\right)/C_{D}$$

where *n_L->S_* is the number of L->S mutation in the Human Gene Mutation Database (HGMD); $P_{L->S}^{j}$ is the PhastCons score of j-th L->S mutation in HGMD. C_D_ is the total number of protein-coding mutations in HGMD. Then, we calculate the CASM score as$h_{L->S,1}/a_{L->S,1}$. For a L->S mutation with PhastCons score of 0, we estimate the CASM score by:

$h_{L->S,0}/a_{L->S,0}=\frac{\left(\sum_{j=1}^{m_{L->S}} f_{j}(1-P_{L->S}^{j})\right)/\left(\sum_{i=j}^{m} f_{j}\right)}{\left(\sum_{j=1}^{n_{L->S}} (1-P_{L->S}^{j})\right)/C_{D}}$.

Finally, for a L->S mutation with PhastCons score of *x* ($0\leq x\leq1$), we heuristically calculate its CASM score by: $\frac{h_{L->S,0}}{a_{L->S,0}}(1-x)+\frac{h_{L->S,1}}{a_{L->S,1}}x$.

In almost all situations, we use the CASM score to predict variant impact rather than using the two estimated likelihoods of variant being damaging and neutral. In VARPRISM, the likelihood ratio test depends only on the ratio of $\Pr(AAS|N)$ and $\Pr(AAS|D)$ but not the exact values of these two probabilities.

**Using transformed CASM scores in fitDNM**

As one of its inputs, fitDNM expects a functional prediction score file for all possible protein coding mutations in a gene. The functional prediction score should be the probability that the mutation affects the function of the protein, and therefore should take values between 0 and 1. In comparison, the CASM score is the ratio of the likelihood that the mutation is damaging divided by the likelihood that it is risk-neutral. In theory, to convert the likelihood ratio to probability, we need to know the prior probability that a mutation is damaging, which can be hard to estimate accurately. We therefore applied the following empirical approach to generate the transformation function. First, we randomly mutated the entire human exome to generate 5000 protein-coding mutations, denoted as Set 1. Then, we randomly selected 5000 mutations from Human Gene Mutation Database, which were mutations classified as damaging to protein functions, denoted as Set 2. For every mutation in the combined set of Set 1 and Set 2, we calculated its CASM score and PolyPhen-2 score. Using the least-square method, we fitted data with the following logit function between these two scores: $P=\frac{1}{1+\exp[-\mu_{0}-\mu_{1}\log(C)]}$, where *P* represents the PolyPhen-2 score and *C* represents the CASM score. The logit transformation has two desirable properties: 1) it ensures that the transformation function is monotonically increasing; 2) it maps the original CASM scores to a scale between 0 and 1. The estimated value for $\mu_{0}$ is 1.722 and for $\mu_{1}$ is 1.047. We then used the transformed CASM scores as the input of fitDNM in the simulations presented in Supplementary Figure 1.

**Supplementary Tables**

Table S1. Autism candidate risk genes identified by VARPRISM at FDR<0.3.

| **gene** | **p-value** | **q-value** | **Pubmed ID of primary reference** | **Evidence for involvement in ASD** |
| --- | --- | --- | --- | --- |
| *CHD8* | 1.0E-07 | 9.3E-04 | 22495309 | De novo mutation detected in autism patients |
| *DYRK1A* | 1.0E-07 | 9.3E-04 | 22495309 | De novo mutation detected in autism patients |
| *SCN2A* | 4.0E-07 | 1.9E-03 | 12610651 | De novo mutation detected in autism patients |
| *GRIN2B* | 4.0E-07 | 1.9E-03 | 21572417 | De novo mutation detected in autism patients |
| *POGZ* | 1.1E-06 | 4.1E-03 | 22495311 | De novo mutation detected in autism patients |
| *SUV420H1* | 3.7E-06 | 1.2E-02 | 22495306 | De novo mutation detected in autism patients |
| *KDM5B* | 1.5E-05 | 4.0E-02 | 25363768 | De novo mutation detected in autism patients |
| *TBR1* | 1.9E-05 | 4.0E-02 | 22495309 | De novo mutation detected in autism patients |
| *KATNAL2* | 1.9E-05 | 4.0E-02 | 22495306 | De novo mutation detected in autism patients |
| *MYH10* | 2.9E-05 | 5.0E-02 | 25356899 | De novo mutation detected in patients with intellectual disability |
| *TCF7L2* | 3.0E-05 | 5.0E-02 | 25363768 | De novo mutation detected in autism patients |
| *TBL1XR1* | 3.2E-05 | 5.0E-02 | 22495309 | De novo mutation detected in autism patients |
| *DSCAM* | 5.0E-05 | 7.2E-02 | 25363768 | De novo mutation detected in autism patients |
| *KDM6B* | 6.0E-05 | 8.0E-02 | 22542183 | De novo mutation detected in autism patients |
| *OR10Z1* | 7.1E-05 | 8.8E-02 | NA | NA |
| *CHD2* | 7.8E-05 | 9.1E-02 | 23708187 | De novo mutation detected in autism patients |
| *WAC* | 9.4E-05 | 1.0E-01 | 25363768 | De novo mutation detected in autism patients |
| *PDCD1* | 1.0E-04 | 1.0E-01 | 23073310 | One of the gene implicated in 2q37-deletion syndrome; symptoms of this Mendelian disorder include intellectual deficiency |
| *MFRP* | 1.0E-04 | 1.0E-01 | 22542183 | De novo mutation detected in autism patients |
| *SLC6A8* | 1.2E-04 | 1.1E-01 | 16601898 | De novo mutation detected in autism patients |
| *FOXP1* | 1.9E-04 | 1.7E-01 | 21572417 | De novo mutation detected in autism patients |
| *ANK2* | 2.1E-04 | 1.8E-01 | 22542183 | De novo mutation detected in autism patients |
| *PPP2R5D* | 2.2E-04 | 1.8E-01 | 25418537 | De novo mutation detected in autism patients |
| *ZC3H4* | 2.3E-04 | 1.8E-01 | NA | NA |
| *ARID1B* | 2.4E-04 | 1.8E-01 | 21448237 | De novo mutation detected in autism patients |
| *KCND3* | 2.7E-04 | 1.9E-01 | 22105621 | A KCND3 haplotype was found to associate with nonverbal communication. |
| *ADNP* | 2.9E-04 | 2.0E-01 | 22495309 | De novo mutation detected in autism patients |
| *KRTAP4-4* | 2.9E-04 | 2.0E-01 | NA | NA |
| *ZNF555* | 3.0E-04 | 2.0E-01 | NA | NA |
| *PTEN* | 3.4E-04 | 2.1E-01 | 11496368 | Rare mutations in PTEN are associated with autistic symptom |
| *CTCF* | 3.5E-04 | 2.1E-01 | 23746550 | De novo mutation in CTCF cause intellectual disability |
| *USP45* | 3.6E-04 | 2.1E-01 | NA | NA |
| *MYO1E* | 3.7E-04 | 2.1E-01 | 24047820 | MYO1E is associated with social communication problem in GWAS |
| *DNMT3A* | 4.0E-04 | 2.1E-01 | 23849776 | De novo mutation detected in autism patients |
| *DIP2A* | 4.0E-04 | 2.1E-01 | 22542183 | *DIP2A* |
| *GLRA2* | 4.0E-04 | 2.1E-01 | 20479760 | Rare variants in GLRA2 are associated with ASD |
| *SYNGAP1* | 4.3E-04 | 2.2E-01 | 23161826 | De novo mutations in SYNGAP1 are associated with nonsyndromic intellectual disability |
| *NCKAP1* | 4.7E-04 | 2.3E-01 | 22542183 | De novo mutation detected in autism patients |
| *MPP6* | 4.8E-04 | 2.3E-01 | 25549968 | Non-synonymous variants in MPP6 are associated ASD |
| *NR3C2* | 4.9E-04 | 2.3E-01 | 25363760 | Identified in TADA analysis of ASD patients |
| *ELAVL3* | 5.3E-04 | 2.3E-01 | NA | NA |
| *PLEKHA8* | 5.4E-04 | 2.3E-01 | 22495309 | De novo mutation detected in autism patients |
| *PTK7* | 5.4E-04 | 2.3E-01 | NA | NA |
| *TSPAN4* | 5.6E-04 | 2.4E-01 | NA | NA |
| *TERF2* | 5.7E-04 | 2.4E-01 | NA | NA |
| *GIGYF1* | 6.1E-04 | 2.5E-01 | 25363768 | De novo mutation detected in autism patients |
| *PHF2* | 6.8E-04 | 2.7E-01 | 22542183 | De novo mutation detected in autism patients |
| *MLL5* | 7.5E-04 | 2.8E-01 | 25284784 | De novo mutation detected in autism patients |
| *TSR2* | 7.6E-04 | 2.8E-01 | 22495309 | De novo mutation detected in autism patients |
| *S100G* | 7.8E-04 | 2.8E-01 | 23966865 | Identified in TADA analysis of ASD patients |
| *AKR1C2* | 7.9E-04 | 2.8E-01 | 26123493 | Disregulated in Protocadherin 19 (with mental retardation symptoms) |
| *SLC6A1* | 7.9E-04 | 2.8E-01 | 22495306 | De novo mutation detected in autism patients |
| *MED13L* | 8.3E-04 | 2.9E-01 | 22542183 | De novo mutation detected in autism patients |
| *BST2* | 8.4E-04 | 2.9E-01 | NA | NA |
| *C2orf42* | 8.8E-04 | 2.9E-01 | NA | NA |
| *GLI4* | 9.1E-04 | 2.9E-01 | NA | NA |
| *DLX3* | 9.5E-04 | 2.9E-01 | NA | NA |
| *NUDT4* | 9.8E-04 | 2.9E-01 | NA | NA |
| *CTNNB1* | 9.8E-04 | 2.9E-01 | 22495309 | De novo mutation detected in autism patients |
| *RIPPLY1* | 1.0E-03 | 2.9E-01 | NA | NA |
| *NTNG1* | 1.0E-03 | 2.9E-01 | 15870826 | De novo mutation detected in autism patients |
| *DCAF4L2* | 1.0E-03 | 2.9E-01 | NA | NA |
| *PAFAH1B2* | 1.0E-03 | 2.9E-01 | NA | NA |
| *TUBGCP4* | 1.0E-03 | 2.9E-01 | 22542183 | De novo mutation detected in autism patients |

Table S2. List of VARPRISM identified candidates in each function category.

| **Categories** | **VARPRISM candidates (FDR<0.3) in the category** |
| --- | --- |
| Chromatin Modifiers | *CHD8, CHD2, ARID1B, KDM6B, PHF2, WAC, DNMT3A, SUV420H1* |
| Embryonic | *DYRK1A, ADNP, MED13L, PHF2, KDM5B, POGZ, DNMT3A, SUV420H1, CTCF, PAFAH1B2, ELAVL3, GLRA2, ZNF555* |
| FMRP Targets | *CHD8, ANK2, GRIN2B, DSCAM, ARID1B, KDM6B, ADNP, MED13L, SCN2A, DIP2A, NCKAP1, MYH10, SYNGAP1, CTNNB1, ZC3H4, PTEN, SLC6A1* |
| De novo mutation genes in intellectual disability | *CHD2, SCN2A, WAC, SYNGAP1, CTNNB1, SLC6A1* |
| De novo mutation genes in Schizoprenia | *SCN2A, POGZ, SYNGAP1* |
| Essential Genes | *CHD8, DYRK1A, DSCAM, ADNP, NCKAP1, FOXP1, MYH10, CTNNB1, DNMT3A, SUV420H1, CTCF, MYO1E, TERF2, PTEN, DLX3* |

Table S3. DAVID results on VARPRISM candidate gene list with FDR<0.3, showing only GO categories with FDR<0.1 by DAVID.

| **GO term** | **p-value** | **Genes** | **Fold Enrichment** | **FDR** |
| --- | --- | --- | --- | --- |
| GO:0016568~chromatin modification | 6.09E-07 | *DNMT3A, TBL1XR1, CHD8, MLL5, CTCF, ARID1B, KDM5B, KDM6B, TERF2, SUV420H1* | 9.680835838 | 9.30E-04 |
| GO:0006325~chromatin organization | 9.76E-07 | *DNMT3A, TBL1XR1, CHD8, MLL5, CHD2, CTCF, ARID1B, KDM5B, KDM6B, TERF2, SUV420H1* | 7.719057994 | 0.001489186 |
| GO:0051276~chromosome organization | 9.04E-06 | *DNMT3A, TBL1XR1, CHD8, MLL5, CHD2, CTCF, ARID1B, KDM5B, KDM6B, TERF2, SUV420H1* | 6.01609056 | 0.013789308 |
| GO:0060070~Wnt receptor signaling pathway through beta-catenin | 3.11E-05 | *TBL1XR1, CHD8, TCF7L2, CTNNB1* | 62.41291811 | 0.047507122 |
| GO:0006355~regulation of transcription, DNA-dependent | 3.35E-05 | *ZNF555, DNMT3A, TBL1XR1, ADNP, NR3C2, CTCF, ARID1B, MED13L, TBR1, TCF7L2, FOXP1, CTNNB1, DLX3, MLL5, CHD8, CHD2, RIPPLY1, KDM5B, KDM6B* | 2.842551121 | 0.05105656 |
| GO:0051252~regulation of RNA metabolic process | 4.53E-05 | *ZNF555, DNMT3A, TBL1XR1, ADNP, NR3C2, CTCF, ARID1B, MED13L, TBR1, TCF7L2, FOXP1, CTNNB1, DLX3, MLL5, CHD8, CHD2, RIPPLY1, KDM5B, KDM6B* | 2.779836259 | 0.069093603 |

**Supplementary Figures**

Figure S1. Power of fitDNM using transformed CASM scores, fitDNM using PolyPhen-2 score and VARPRISM in simulations. Left: Power benchmark using *de novo* mutations in ASD risk genes as damaging mutations. Right: Power benchmark using Human Gene Mutation Database (HGMD) variants as damaging mutations. The sample size is 5000 genomes and the number of trials is 1000. We set the statistical significance threshold at 5x 10^-4^.

Figure S2. Average estimate of r (relative damaging mutation rate) in the power analysis.

Figure S3. Histogram of p-values for the 8 genes with *de novo* mutations in the Congenital Heart Disease dataset.

1. Hu H, Huff CD, Moore B, Flygare S, Reese MG, Yandell M: **VAAST 2.0: Improved Variant Classification and Disease-Gene Identification Using a Conservation-Controlled Amino Acid Substitution Matrix.** *Genet Epidemiol* 2013, **37:**622-634.

2. Siepel A, Bejerano G, Pedersen JS, Hinrichs AS, Hou M, Rosenbloom K, Clawson H, Spieth J, Hillier LW, Richards S, et al: **Evolutionarily conserved elements in vertebrate, insect, worm, and yeast genomes.** *Genome Res* 2005, **15:**1034-1050.
